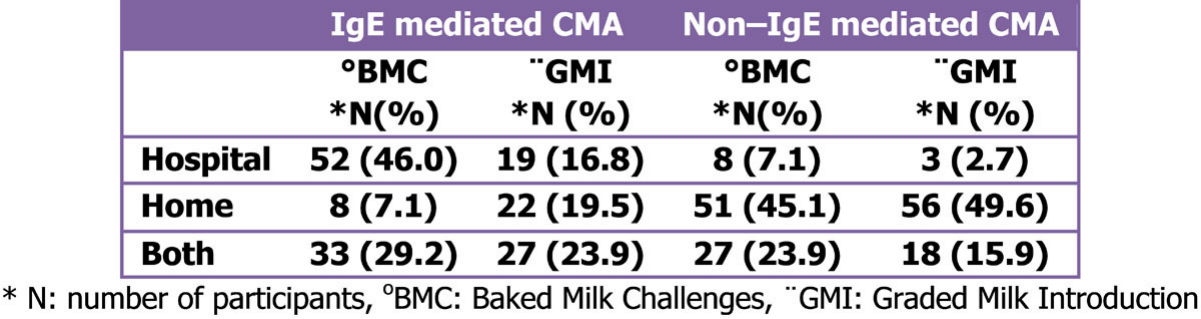# Use of baked milk challenges in clinical practice: a worldwide survey

**DOI:** 10.1186/2045-7022-5-S3-P148

**Published:** 2015-03-30

**Authors:** Yiota (Panagiota) Athanasopoulou, Tara Dean², Carina Venter

**Affiliations:** 1 School of Health Sciences and Social Work, University of Portsmouth, Portsmouth, United Kingdom; 2 The David Hide Asthma and Allergy Research Centre, Isle of Wight, United Kingdom

## Background

Current research suggests that graded introduction of cow's milk, starting with baked milk, may be used as a prognostic indicator for outgrowing Cow's Milk Allergy (CMA).

## Aim

This survey aimed to investigate the attitudes and practice of health professionals on the use of baked milk challenges and graded introduction of milk products in IgE and non-IgE mediated CMA in different regions of the world.

## Method

The participants were identified by National and International Health Professional Associations and completed the survey online.

## Results

Of 113 participants 51(45.1%) were dietitians, 31(27.4%) Paediatric Allergists and Paediatricians, 15(13.3%) Allergists/Clinical Immunologists, 2(1.9%) General Practitioners, and 14(12.4%) other health scientists. 14(12.7%) of participants reported that they use baked milk challenges to confirm the diagnosis in CMA and 82(73.2%) to determine tolerance to milk. 52(46%) perform baked milk challenges in IgE mediated CMA in hospital, 8(7.1%) suggest home and 33(29.2%) in both places. In non-IgE mediated CMA, 8(7.1%) conduct these challenges in hospital, 51(45.1%) at home and 27(23.9%) at both places. 17(15%) of responders stated that they use graded introduction of milk containing foods (Milk Ladder) to diagnose CMA and 80(70.8%) to determine tolerance. 19(16.8%) carry out the Milk Ladder in IgE CMA in hospital, 22(19.5%) use it at home and 27(23.9%) in both places. 3(2.7%) perform graded milk introduction in non-IgE mediated CMA in hospital, 56(49.6%) suggest home, and 18(15.9%) in both places.

## Conclusion

Data indicates that most of the health care professionals use baked milk challenges and graded introduction of baked milk foods to determine the development of tolerance to cow's milk in clinical practice.

**Figure 1:**